# Dynamic change of the systemic immune inflammation index is a risk factor for patients with oropharyngeal cancer: a case control study and an additional HPV-status subgroup analysis

**DOI:** 10.1186/s40001-023-01157-9

**Published:** 2023-06-23

**Authors:** XiaoChuan Gan, QiTao Gou, Jing Zhu, Tao Zhang

**Affiliations:** Department of Oncology, The First Affiliated Hospital of Chongqing Medical University, 1 Youyi Road, Yuzhong, Chongqing, 400016 China

**Keywords:** Dynamic change, Inflammation, Prognosis, Human papillomavirus, Serial monitoring, Oropharyngeal cancer

## Abstract

**Background:**

The study aimed to analyze the relationship between the dynamic systemic immune inflammation index (SII), human papillomavirus (HPV) infection, and the prognosis of oropharyngeal cancer patients.

**Method:**

We retrospectively obtained the data for 131 patients treated with curative treatments and calculated their SII values based on results acquired approximately 9 months after the first treatment. The entire cohort was divided into groups according to dynamic SII and HPV infection, and their prognoses were compared.

**Results:**

The high SII group, particularly the persistently high SII group, had a poor prognosis, and static SII levels cannot fully reflect the prognosis of patients with oropharyngeal cancer. In HPV− patients, unfavorable dynamic SII and the site of tumor locating at the tongue base were all significantly associated with decreased disease-free survival. In contrast, no characteristic was presented as a poor prognostic factor for disease-free or overall survival in HPV+ patients.

**Conclusion:**

Dynamic SII values are more comprehensive prognostic indicators for oropharyngeal cancer patients, particularly HPV− patients. It could imply that an HPV− oropharyngeal cancer patient who experienced unfavorable dynamic changes in SII should receive more frequent tests or more advanced therapies.

## Introduction

Oropharyngeal cancer (OPC) is a type of squamous cell carcinoma of the head and neck (SCCHN) that refers to malignant tumors in the tonsil, tongue base, soft palate, and uvula, among other places [1]. In 2018, nearly 93,000 patients were diagnosed with OPC, accounting for more than 13% of all head and neck cancers worldwide [2].

SCCHN, including oropharyngeal carcinoma, may be linked to common risk factors, such as alcohol consumption, T3/4 stage, vascular invasion, external capsule invasion, margin positivity, and human papillomavirus (HPV) infection [1–8]. Besides, growing evidence indicates that inflammation is another adverse factor for cancers [9]. Some simple parameters derived from routine complete blood count (CBC) include neutrophils, lymphocytes, monocytes, platelets (PLT), and various blood cell ratios [10–12]––have been found to predict the prognosis in patients with head and neck malignancies [13, 14]. Furthermore, the systemic immune inflammation index (SII), calculated from CBC by multiplying the absolute neutrophil and platelet counts and then dividing by the absolute lymphocyte count, has a significant impact on the survival rate of patients with certain solid tumors, such as head and neck carcinomas [10–16], pancreatic cancers [17], gastrointestinal cancers [18], cervical cancers [19], bladder cancers [20]. However, previous studies [15, 16] focused on the relationship between the baseline SII, or the SII measured at only two time points before and after treatment, and prognosis [4]. Given that OPC progression is a multistep and complex process, dynamic change and serial monitoring of the SII values may provide better measures to predict OPC progression than static SII levels. Therefore, the dynamic change of SII values may provide a more accurate alternative for predicting the prognosis of OPC patients.

In this study, we aim to improve the prognostic value of SII by sequentially monitoring its dynamic change in OPC patients and comparing the mixed effects of various clinical or pathological risk factors.

## Materials and methods

### Patients

**Fig. 1:**
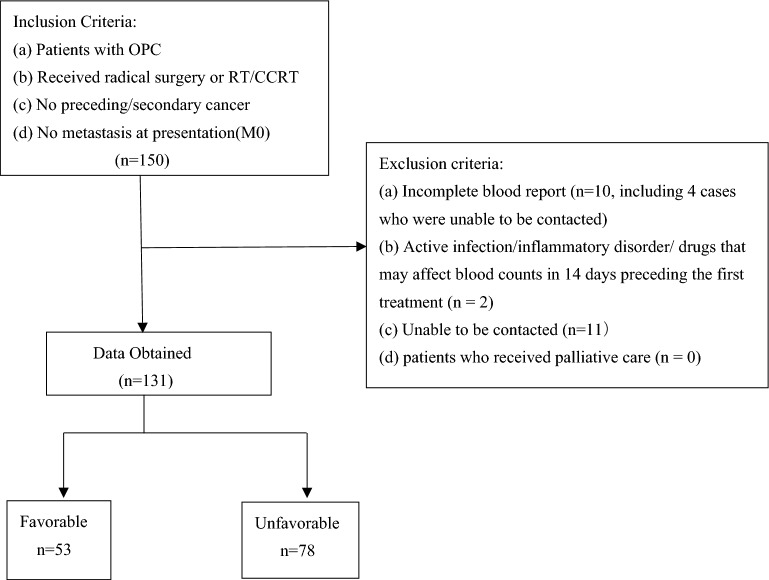
Flow of patients through the study. OPC, oropharyngeal cancer; RT, radiotherapy; CCRT, chemoradiotherapy

We evaluated 131 patients diagnosed with OPC who received curative treatments in the first affiliated hospital of Chongqing Medical University from January 1st, 2013 to November 30th, 2021. The following were the inclusion criteria: (a) patients with a histopathological diagnosis of OPC; (b) patients who received curative radiotherapy/chemoradiotherapy (RT/CCRT); (c) no history of secondary cancer diagnosed in the 3 years preceding or following OPC treatments; and (d) no metastasis at presentation (M0). The following were the exclusion criteria: (a) incomplete laboratory blood reports (*n* = 10, including four cases that could not be contacted); (b) a history of an active infectious, inflammatory, or oral administration of drugs that could affect routine blood counts in the 14 days preceding the first treatment for OPC (*n* = 2); (c) patients who could not be contacted (*n* = 11); (d) patients who received palliative care (*n* = 0). The flow of patients through the study is depicted in Fig. 1.

### Data collection

Considering the reference index of HPV was not included in this study, patients were staged using the 7th edition of the American Joint Committee on Cancer (AJCC) TNM classification system [21]. Patients’ post-treatment monitoring included routine clinical and laboratory examinations and regular imaging scans to monitor recurrence, metastasis, and death. Age, gender, tumor site, stage, T stage, N stage, positive margins, extranodal extension, muscular or capsule invasion, nerve invasion, vascular invasion, cigarette smoking, alcohol drinking, and the dynamic change of SII were the basic clinical features of the patients. Peripheral venous whole blood samples were collected 1–2 weeks before the first treatment and 1 month, 3 months, 5 months, and 8 months later. Finally, we created a serial monitoring curve using data from 131 patients who had all blood tests completed at the required five time points before and after the first treatment.

### Immunohistochemical detection

When performing subgroup analysis, we conduct immunohistochemical detection on tumor tissues of OPC patients diagnosed after 2018. Thus, 57 out of 131 people’s formalin-fixed paraffin-embedded tumor samples were examined for high-risk HPV (HR-HPV) infection using immunohistochemical assessment of P16 (INK4A) protein expression. Immunostainings were performed on 3-μm formalin-fixed, paraffin-embedded tissue sections. Endogenous enzymes were inhibited by hydrogen peroxide and PBS after dewaxing with xylene and alcohol. By boiling in citrate buffer, antigens were extracted. The p16 (Abcam, MX007) primary antibodies were used. Furthermore, secondary detection was carried out with the goat anti-mouse/rabbit IgG Polymer III. They were then colored and observed under microscopes. According to the AJCC/UICC 8th Edition New Staging Rules for p16 detection in OPC, diffuse p16 in nucleus and cytoplasm staining of ≥ 75% and at least moderate staining intensity is classified as p16 positivity [22]. The stages of these 57 OPC patients were classified using the American Joint Committee on Cancer (AJCC) TNM classification system, 8th edition [23].

### Follow-up

Considering sensitivity and specificity, as well as following a time-to-progression approach, a receiver operating characteristic (ROC) curve for progression, including local recurrence, local metastasis, and distant metastasis, or death, was drawn. The area under the curve (AUC) of SII was calculated to be 0.662, 95% CI 0.569–0.756 (*p* = 0.01), and the Youden’s index was used to estimate the optimal cutoff value for SII (706.04 in our study). Therefore, SII ≥ 706.04 and < 706.04 were called high or low SII, respectively. All members were classified into the favorable group (*n* = 53) and the unfavorable group (*n* = 78). The favorable group included Category 1 (persistently low SII level) (*n* = 24) and Category 2 (high to low SII level) (*n* = 29), and the unfavorable group included Category 3 (persistently high SII level) (*n* = 36) and Category 4 (low-to-high SII level) (*n* = 42). If the patients whose the five SII measurements (one prior to treatment and four thereafter) were all less or larger than 706.04, they were classified into Category 1 (persistently low SII level) and Category 3 (persistently high SII level), respectively. If SII levels at the required five time points were not all less or larger than 706.04, then the five SII measurements of this part of the patients were made into a line chart in which SII levels changed with time, so as to observe the trend of the lines. If the trend of lines was continuously downward, this part of the patients were classified into Category 2 (high to low SII level); if the trend continued to rise, they were classified into Category 4 (low-to-high SII level). Furthermore, the first and last SII values were compared for patients whose SII levels fluctuated repeatedly during these five times. If the patients in whom these two SII measurements were all less or larger than 706.04, they were classified into Category 1 and Category 3, respectively. If not, the patients with SII > 706.04 for the first time and SII < 706.04 for the last time were classified into Category 2, otherwise they were classified into Category 4. Two authors independently used the above classification method to classify each patient, and cross-checked the results. According to the optimal cutoff value for SII in this study, the same method is used to divide the two different HPV-infection-status groups, respectively, into two groups (the favorable group and unfavorable group) or four groups (Category 1–4). All subjects were followed up regularly until recurrence, metastasis, death, or study termination. In this retrospective study, two endpoints were considered. The primary endpoint was disease-free survival (DFS) (time from first treatment to progression, including local recurrence, local metastasis, and distant metastasis, or death), and the secondary endpoint was overall survival (OS) (time from first treatment to death). Different groups’ clinical characteristics, DFS and OS, were compared.

### Statistical analysis

Data were statistically analyzed using SPSS 23.0 software. General patient characteristics (such as age and gender) and clinical information (such as TNM classification, tumor site, vascular invasion, progression and death) are expressed as a frequency and percentage. To compare the DFS and OS of patients in the two groups, survival analysis was performed using the Kaplan–Meier method and the log-rank test. The mean and standard deviation (SD) described normally distributed data, whereas the median and interquartile range were expressed for non-normally distributed data. In the comparison between groups, quantitative data were analyzed by the *T*-test (normally distributed data) or the nonparametric rank-sum test (non-normally distributed data), and the Chi-square test was employed for categorical data. In univariate and multivariate Cox proportional hazard analysis, each classification in multiple classification variables was compared with the first classification. To identify independent factors that influence prognosis, a multivariate Cox proportional hazards regression model analysis was performed on clinical factors that were statistically significant in univariate analysis. Two-tailed *p* values < 0.05 were considered statistically significant.

## Results

### Patients’ characteristics

Patients included 94 (71.8%) men and 37 (28.2%) women with a combined median age of 59.72 years (range 25–82 years), and 53 (40.5%) and 78 (59.5%) were in the favorable or unfavorable groups, respectively. Primary tumors were found in the tonsil in 64 (48.9%) patients, the tongue base in 56 (42.7%) of patients, and other sites (including the pharyngolaryngeal fossa, the soft palate and the uvula) in 11 (8.4%) of patients. Of the 131 patients, 23 (17.6%) were in stages I and II, while 108 (82.4%) were in stages III and IV. The median follow-up time was 30 months for the total population. The disease-free survival (DFS) at 3 years was 28%, and The 3-year OS for OPC patients was 59%. Table 1 displays the clinical characteristics of the 131 patients.

**Table 1:** The correlation between clinical characteristics and dynamic change of the SII

Variables	Favorable (*n* = 53)	Unfavorable (*n* = 78)	Total (*n* = 131)	*X*^2^ or *Z*	*p*-value
Age, median (P25, P75)	56 (51.5, 66.5)	61 (51.0, 70.5)		1.304*	0.192
Gender, *n* (%)					
Female	15 (28.3)	22 (28.2)	37	0.000^#^	1.000
Male	38 (71.7)	56 (71.8)	94		
Nerve invasion (*n*, %)					
No	52 (98.1)	74 (94.9)	126	0.236^#^	0.627
Yes	1 (1.9)	4 (5.1)	5		
Vascular invasion (*n*, %)					
No	51 (96.2)	70 (89.7)	121	1.074^#^	0.300
Yes	2 (3.8)	8 (10.3)	10		
Cigarette smoking (*n*, %)					
No	22 (41.5)	31 (39.7)	53	0.041^#^	0.858
Yes	31 (58.5)	47 (60.3)	78		
Alcohol drinking (*n*, %)					
No	26 (54.7)	25 (44.9)	59	0.581^#^	0.478
Yes	27 (50.9)	45 (57.7)	72		
Tumor site (*n*, %)					
Tonsil	29 (54.7)	35 (44.9)	64	5.128^#^	0.077
Tongue base	23 (43.4)	33 (42.3)	56		
Other^+^	1 (1.9)	10 (12.8)	11		
Positive margin (*n*, %)					
No	14 (26.4)	26 (33.3)	40	1.202^#^	0.570
Yes	1 (1.9)	3 (3.8)	4		
No-operation	38 (71.7)	49 (62.8)	87		
Extranodal extension (*n*, %)					
No	13 (24.5)	23 (29.5)	36	0.560^#^	0.756
Yes	4 (7.5)	7 (9.0)	11		
No-operation	36 (67.9)	48 (61.5)	84		
Muscular/capsule invasion (*n*, %)					
No	6 (11.3)	21 (26.9)	27	4.696^#^	0.096
Yes	10 (18.9)	12 (15.4)	22		
No-operation	37 (69.8)	45 (57.7)	82		
Stage (*n*, %)					
I–II	10 (18.9)	13 (16.7)	23	0.106^#^	0.817
III–IV	43 (81.1)	65 (83.3)	108		
T stage (*n*, %)					
I–II	27 (50.9)	38 (48.7)	65	0.063^#^	0.806
III–IV	26 (49.1)	40 (51.3)	66		
N stage (*n*, %)					
N0	17 (32.1)	24 (30.8)	41	0.025^#^	1.000
N+	36 (67.9)	54 (69.2)	90		
Progression (*n*, %)					
No	26 (49.1)	23 (29.5)	49	5.162^#^	0.028
Yes	27 (50.9)	55 (70.5)	82		
Death (*n*, %)					
No	35 (66.0)	37 (47.4)	72	4.411^#^	0.049
Yes	18 (34.0)	41 (52.6)	59		

^+^Including the pharyngolaryngeal fossa, the soft palate and the uvula; *Z-value; ^#^Chi-square value. Both groups were comparable regarding these clinical characteristics including age, sex, nerve invasion, vascular invasion, cigarette smoking, alcohol drinking, positive margin, extranodal extension, muscular or capsule invasion, stage, T stage, N stage

### Value of an unfavorable dynamic change of the SII as an independent indicator for OPC progression or death

Table 1 lists the difference in clinicopathological features between the favorable and the unfavorable groups. Both groups were well balanced for the prognostic factors. Members of the unfavorable group were found to be more likely to have positive events (progression or death) (*p* = 0.028) and more likely to die (*p* = 0.049). However, other baseline clinical characteristics showed no statistically significant difference (*p* > 0.05).

**Fig. 2:**
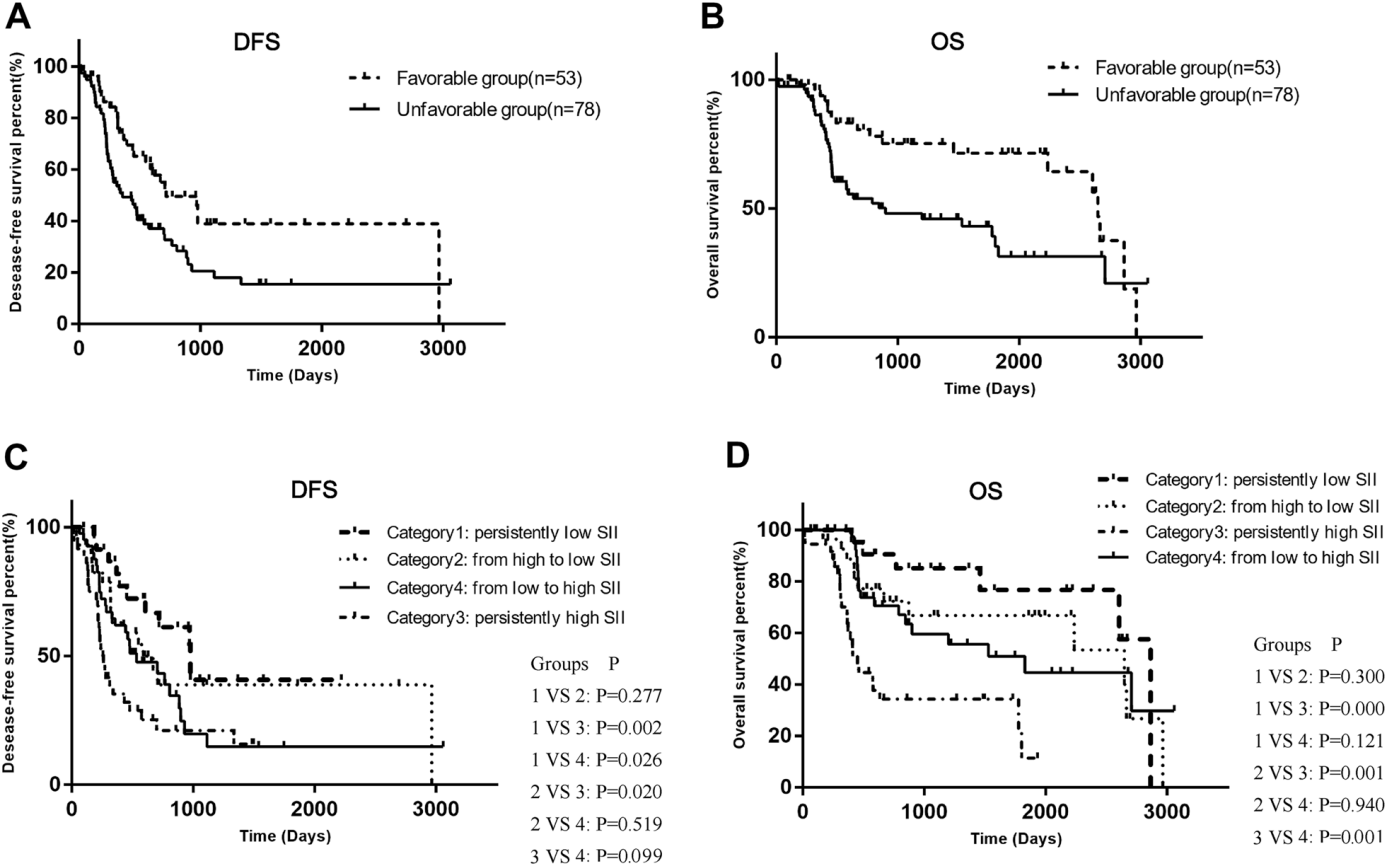
The dynamic change of the SII associate significantly with progression or death. **A** Kaplan–Meier analysis for time of DFS in OPC patients with unfavorable and favorable groups. **B** Kaplan–Meier analysis for time of OS in OPC patients with unfavorable and favorable groups. **C** Kaplan–Meier analysis for time of DFS in OPC patients among Category 1–4. **D** Kaplan–Meier analysis for time of OS in OPC patients among Category 1–4

According to Kaplan–Meier analysis, the difference in DFS between the unfavorable (median DFS 359.0 days, 95% CI 176.3–541.7) and favorable (median DFS 719.0 days, 95% CI 431.3–1006.7) groups was statistically significant (*p* = 0.006) (Fig. 2A). Similarly, there was also a statistically significant difference in OS between the unfavorable group (median of OS 899.0 days, 95% CI 2567.5–1824.3) and the favorable group (median of OS 2648.0 days, 95% CI 2567.5–2728.5) with *p* = 0.008 (Fig. 2B).

**Fig. 3:**
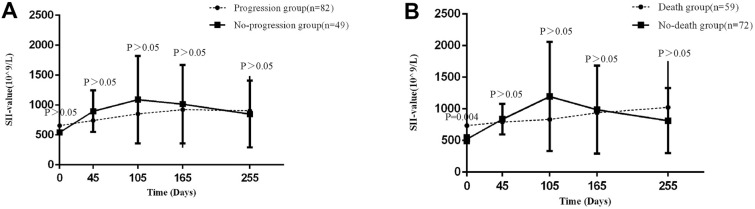
The serial monitoring of the SII. **A** The serial monitoring of the SII discriminate for patients with high profession risks. **B** The serial monitoring of the SII discriminate for patients with high death risks

The prognostic significance of SII was then thoroughly investigated. All members were classified into the following groups: Category 1, persistently low SII level (*n* = 24); Category 2, high to low SII level (*n* = 29); Category 3, persistently high SII level (*n* = 36); and Category 4, low-to-high SII level (*n* = 42). The four categories are depicted in Fig. 3. For DFS, the *p*-values were 0.277, 0.002, 0.026, 0.020, 0.519, and 0.099 for Categories 1 and 2, Categories 1 and 3, Categories 2 and 3, Categories 2 and 4, and Categories 3 and 4, respectively (Fig. 2C). Among them, members of Category 1 had the longest time of DFS (median of DFS 978.0 days, 95% CI 677.8–1278.2). However, the difference between the DFS of Category 1 and Category 2 (median of DFS 589.0 days, 95% CI 285.1–892.9) is not statistically significant (*p* > 0.05). Furthermore, patients in Category 3 had the shortest DFS (median DFS 257.0 days, 95% CI 196.7–317.3), but there was no statistically significant difference when compared to patients in Category 4 (median of DFS 533.0 days, 95% CI 238.3–827.7) (*p* > 0.05). Moreover, for OS, the *p*-values were as follows: 0.300, 0.000, 0.121, 0.001, 0.940, and 0.001 for Categories 1 and 2, Categories 1 and 3, Categories 1 and 4, Categories 2 and 3, Categories 2 and 4, and Categories 3 and 4, respectively (Fig. 1D). The time of OS in Category 1 was the longest (median of OS 2648.0 days, 95% CI 591.9–4704.1), but there was no significant difference between OS in Category 1 and OS in Category 2 (median of OS 2353.0 days, 95% CI 1915.8–2987.6) (*p* > 0.05). Similarly, the time of OS in Category 3 was the shortest with a median of 450.0 days, 95% CI 356.2–543.8). Surprisingly, there was a significant difference compared to Category 4 (median of OS 1828.0 days, 95% CI 697.3–2958.7) (*p* = 0.001), indicating that the SII’s dynamic change may serve as a more powerful index.

### Factors associated with recurrence and their progression or death significance

Prognostic indicators were determined using the Cox proportional hazard model. In univariate analysis of DFS, the following variables were found to be discriminating: dynamic change of the SII (HR 1.903, 95% CI 1.197–3.025, *p* = 0.007), the tumor site is tongue base compared to the tonsil (HR, 1.880; 95% CI 1.186–2.981, *p* = 0.007), stage (HR 2.085, 95% CI 1.003–4.338, *p* = 0.049), T stage (HR 1.621, 95% CI 1.045–2.514, *p* = 0.031) and alcohol drinking (HR, 1.583; 95% CI 1.008–2.487, *p* = 0.046) (Table 2). Moreover, these discriminating variables were further evaluated in multivariate analysis. Multivariate analysis revealed that in comparison with the tonsil, the tumor located at the tongue root (HR 2.058; 95% CI 1.255–3.374, *p* = 0.004), stage (HR 1.927; 95% CI 1.055–4.343, *p* = 0.039), alcohol drinking (HR 1.656; 95% CI 1.030–2.663, *p* = 0.037) were independent indicators for predicting OPC DFS. Furthermore, the SII dynamic change served as an independent indicator (HR 1.770, 95% CI 1.100–2.848; *p* = 0.019) (Table 2).

**Table 2:** Univariate and multivariate Cox proportional hazard analysis of factors associated with DFS and OS

Variables	DFS				OS			
	Univariate analysis		Multivariate analysis		Univariate analysis		Multivariate analysis	
	HR (95% CI)	*p*-value	HR (95% CI)	*p*-value	HR (95% CI)	*p*-value	HR (95% CI)	*p*-value
Dynamic SII	1.90 (1.20–3.03)	0.007*	1.77 (1.10–2.85)	0.019*	2.11 (1.20–3.69)	0.009*	2.01 (1.13–3.59)	0.018*
Age	0.99 (0.97–1.01)	0.349			1.01 (0.99–1.03)	0.400		
Gender	1.28 (0.76–2.17)	0.351			0.93 (0.52–1.6)	0.808		
Tumor site								
Tonsil$		0.103				0.320		
Pharyngolaryngeal fossa	0.75 (0.10–5.51)	0.779			0.00 (0.00–0.00)	0.978		
Soft palate	1.41 (0.55–3.63)	0.474			1.72 (0.64–4.60)	0.282		
Tongue base	1.88 (1.19–2.98)	0.007*	2.06 (1.26–3.37)	0.004*	1.80 (1.02–3.18)	0.043*	1.99 (1.12–3.55)	0.019*
Uvula	1.93 (0.26–14.21)	0.518			2.83 (0.38–21.18)	0.312		
Stage	2.09 (1.00–4.34)	0.049*	1.93 (0.86–4.34)	0.039*	1.40 (0.63–3.08)	0.410		
T stage	1.62 (1.05–2.51)	0.031*	1.13 (0.69–2.51)	0.113	1.45 (0.86–2.43)	0.163		
N stage	1.12 (0.69–1.80)	0.645			1.97 (0.57–2.67)	0.922		
Positive margin	1.95 (0.75–2.70)	0.650			1.05 (0.79–1.39)	0.735		
Extranodal extension	1.91 (0.37–2.51)	0.435			1.00 (0.75–1.33)	0.990		
Muscular or capsule invasion	1.03 (0.92–1.20)	0.556			1.08 (0.71–1.35)	0.894		
Nerve invasion	1.25 (0.30–5.13)	0.760			1.27 (0.11–5.59)	0.794		
Vascular invasion	1.84 (0.79–4.28)	0.157			4.07 (1.69–9.79)	0.002*	4.22 (1.69–10.49)	0.002*
Cigarette smoking	1.42 (0.90–2.24)	0.136			1.19 (0.69–2.05)	0.524		
Alcohol drinking	1.58 (1.01–2.49)	0.046*	1.66 (1.03–2.66)	0.037*	1.31 (0.77–2.22)	0.316		

*B*, coefficient; SE, standard error; Wald, statistic; HR, hazard ratio; CI, confidence interval

*Significant different

^$^Dummy argument

Furthermore, in univariate analysis of OS, the following variables were found to be discriminating: dynamic change of the SII (HR, 2.107; 95% CI 1.203–3.689, *p* = 0.009), tumor site tongue base versus tonsil (HR, 1.799; 95% CI 1.019–3.175, *p* = 0.043), and vascular invasion (HR, 4.068; 95% CI 1.691–9.788, *p* = 0.002) (Table 2). Then, these discriminating variables were further evaluated in multivariate analysis, which shows that the dynamic change of the SII (HR 2.012; CI 1.127–3.592, *p* = 0.018), the tumor locating at the tongue base (HR 1.994; 95% CI 1.119–3.551, *p* = 0.019), and vascular invasion (HR 4.215; 95% CI 1.694–10.491, *p* = 0.002) are independent indicators for predicting OPC OS (Table 2).

### The role of serial monitoring in forecasting progression

In this study, 131 patients completed routine blood tests for approximately 9 months after the first treatment, almost including their SII status before, during, and after the entire therapy process. SII was measured 1–2 weeks before the first treatment (operation/chemotherapy/radiotherapy) and 1 month (SII2), 3 months (SII3), 5 months (SII4) and 8 months (SII5) after the first treatment. The entire cohort was divided into progression and no-progressive groups, no-death and death groups, relative to whether the patients had positive events (recurrence, local metastasis, distant metastasis, or death) or whether the patients had died. The findings revealed no statistically significant difference in SII between the no-progression and progression groups at any time, whether before or after treatment. (*p* > 0.05) (Fig. 3A). However, the differences in SII between the no-death and death groups were statistically significant at one point: SII1 *p* = 0.004, SII2 *p* > 0.05, SII3 *p* > 0.05, SII4 *p* > 0.05, SII5 *p* > 0.05 (Fig. 3B).

### Subgroups: HPV+, HPV−

**Fig. 4:**
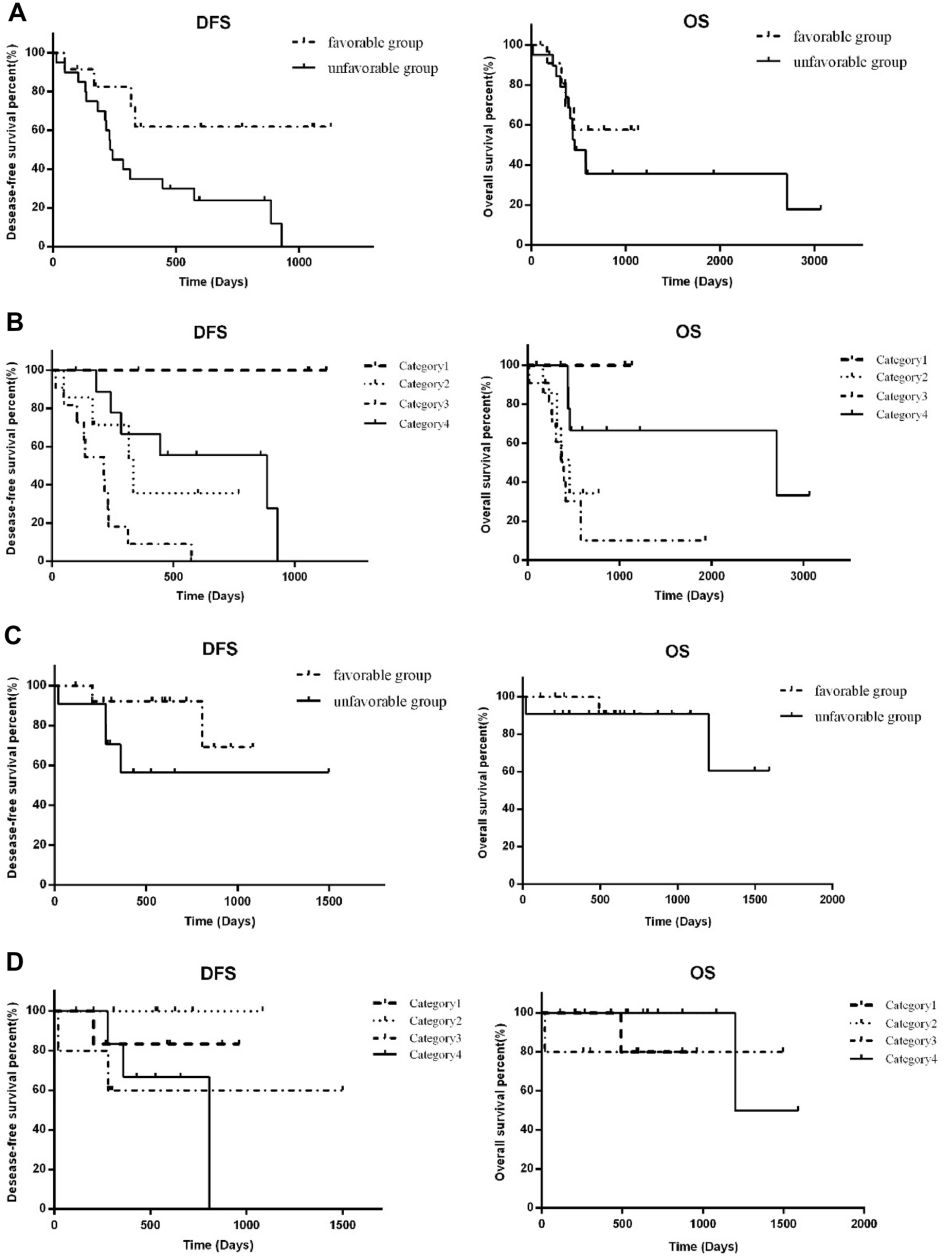
The dynamic change of the SII and HPV(P16) status associate significantly with progression or death. **A** Kaplan–Meier analysis for time of DFS and OS in HPV(−) OPC patients with favorable and unfavorable groups. **B** Kaplan–Meier analysis for time of DFS and OS in HPV(−)OPC patients among Category 1–4. **C** Kaplan–Meier analysis for time of DFS and OS in HPV(+) OPC patients with favorable and unfavorable groups. **D** Kaplan–Meier analysis for time of DFS and OS in HPV(+)OPC patients among Category 1–4

OS and DFS based on HPV status were presented using univariate (UVA) and multivariate Cox analysis (MVA). The favorable HPV− subgroup had significantly longer DFS than the unfavorable HPV− subgroup (*p* = 0.018), but there was no significant difference in OS between the two subgroups (*p* > 0.05) (Fig. 4A). In both SII subgroups of HPV+ patients, DFS and OS were not markedly different (*p* > 0.05, *p* > 0.05, respectively) (Fig. 4C).

Then, we classify all members into four categories. In HPV− patients, the p-values for DFS were as follows: 0.056, 0.002, 0.019, 0.043, 0.382, and 0.001 for Categories 1 and 2, Categories 1 and 3, Categories 1 and 4, Categories 2 and 3, Categories 2 and 4, and Categories 3 and 4, respectively (Fig. 4B). Similarly, for OS, the *p*-values were observed for Categories 1 and 2 (0.078), Categories 1 and 3 (0.014), Categories 1 and 4 (0.286), Categories 2 and 3 (0.444), Categories 2 and 4 (0.125), and Categories 3 and 4 (0.006), respectively (Fig. 4B). Therefore, same as the total 131 samples, there was a significant difference in DFS and OS between Categories 3 and 4 (*p* = 0.001 and *p* = 0.006, respectively), indicating that patients with persistently high SII (Category 4) may have a poorer prognosis. While in HPV+ patients, there was no statistical difference in DFS or OS between any two subgroups (*p* > 0.05) (Fig. 4D).

In UVA, poor prognostic factors for DFS in HPV− patients were as follows: unfavorable dynamic SII (HR 3.52, 95% CI 1.16–10.68, *p* = 0.026), and the site of tumor locating at tongue base (HR 2.99, 95% CI 1.03–8.70, *p* = 0.044). Unfavorable dynamic SII (HR 3.68, 95% CI 1.20–11.32, *p* = 0.023) and tumor location at tongue base (HR 2.98, 95% CI 1.04–8.60, *p* = 0.043) were both independent poor prognostic factors for DFS in MVA. In terms of OS, age (HR 1.08, 95% CI 1.02–1.14, *p* = 0.009) and tumor location at the tongue base (HR 4.14, 95% CI 1.11–15.41, *p* = 0.034) were both associated with significant decreases in HPV− patients. Furthermore, multivariate analysis showed that age was an independent prognostic factor (HR 1.07, 95% CI 1.02–1.13, *p* = 0.012) for OS. The location of the tumor at the base of the tongue (HR 3.95, 95% CI 0.99–15.72, *p* = 0.051) marginally independently predicted poorer OS in HPV− patients. In HPV+ patients, no characteristic was found to be a poor predictor of DFS or OS.

## Discussion

The SII is a clinically objective, readily available biological prognostic indicator for patients with cancers [15–20]. The study found that the SII has a more comprehensive prognostic evaluation value for patients with OPC because it involves more singular parameters (neutrophils, lymphocytes, and platelets) simultaneously than the others (neutrophil to lymphocyte ratio, platelet lymphocyte ratio), implying that the SII will better reflect the systemic immune response [4]. Therefore, we conducted a clinical retrospective study to evaluate the prognostic value of dynamic change in SII in patients with OPC. The research noted that some singular parameters (neutrophils, lymphocytes, and platelets) were significantly poor prognostic factors for survival and disease control in the HPV+ OPC group. However, no correlation was observed in the HPV− group [10]. Another study [4] showed different pre-treatment and post-treatment parameters predicting OS and DFS in HPV− and HPV+ patients. The author reported that HPV+ patients with higher pre-treatment NLR and SII had both inferior OS and DFS. However, there was no correlation in the HPV− group, similar to Huang’s study of 510 adults [10]. It confirms that inflammatory mediators significantly influence treatment outcomes, especially in HPV+ OPC individuals [4]. In terms of the mechanism, some studies have suggested that neutrophils promote cancer cells invasion, proliferation and metastasis by releasing chemokines [24–30], and neutrophils also help increase the adhesion of tumor cells to endothelium as well as their ability to plant at distant sites [31–33]. Platelet growth factor production protects malignant cells from natural killer cell-induced cell death [34] and blunted lymphocyte-mediated immune response against malignant cells [35].

It is important to note that Huang et al. only compared baseline peripheral blood indexes [10]. Likewise, Adam Brewczyński et al. [4] measured and compared SII levels twice—before and after treatment, considering that the progression of oropharyngeal cancer is a multistep and complex process, and the response of each individual to the treatment is different, in addition, each individual’s response to treatment is unique; additionally, the stress state or even infection will be caused by the toxic side effects of surgery, radiotherapy, or chemotherapy, resulting in varying degrees of deviation from SII values. Thus, SII monitoring requires a longer follow-up period. We measured and tracked dynamic SII changes in our study for about 9 months. We found that the DFS and OS of patients in the unfavorable group were markedly lower than the patients in the favorable group. However, the persistently high SII group and from low-to-high SII group both belong to the unfavorable group. The median OS of the persistently high SII group is significantly lower than that of the low-to-high SII group, indicating that the persistently high SII group’s pathophysiology provided a more favorable environment for tumor growth, infiltration, and metastasis. Furthermore, there was no significant correlation between static SII levels and disease progression or death at any of the five time points, indicating that, when compared to static SII values, dynamic SII change may provide a more comprehensive means of predicting the prognosis of oropharyngeal cancer.

In addition to the dynamic change of SII, we discovered that the following variables were retained in the multivariable model as independent risk factors for DFS: tumor location at the tongue root (HR 2.058, *p* = 0.004), stage (HR 1.927, *p* = 0.039), and alcohol consumption (HR 1.656, *p* = 0.037). On the other hand, tumor location at the tongue base (HR 1.994, *p* = 0.019) and vascular invasion (HR 4.215, *p* = 0.002) were independently associated with OS.

When we classified patients with different HPV status into favorable and unfavorable groups by following up on SII for longer, our results were inconsistent with the previously reported study [4]. In HPV− patients, a favorable change of SII may predict superior DFS, and the persistently high SII group had inferior DFS and OS than the low-to-high SII group. However, dynamic SII did not show any relation in the HPV+ group. Moreover, Brewczyński et al. [4] found the value for the optimal cutoff of SII in their study is 448.60, while our study showed a value for the optimal cutoff of SII is 706.04. This difference may be related to differences in treatment method and dose, patient compliance, individual physical fitness level and nutritional status, blood sample test reagents and sample size. Therefore, a large sample size is required to determine the intercept point.

Our study had the following limitations: (1) there are insufficient subjects with p16 IHC data; (2) the duration of the study was insufficient to detect early progression, let alone death; (3) the imaging methods failed to detect the first recurrence; (4) each patient agreed to a different treatment plan; (5) the time available for monitoring SII is still insufficient because if SII continues to rise over a given period of time, more frequent tests or advanced treatment are required; (6) the recall deviation was relatively large because it was a retrospective study. Larger prospective studies must address these limitations to validate the predictive value of serial SII monitoring (7) HPV genotyping cannot be taken into account in this study because of the limited funds of this agency. There was mounting evidence that HPV type was crucial, with HPV33-positive patients reporting worse prognosis than HPV-negative ones [36]. On the other hand, according to the National Comprehensive Cancer Network (NCCN) guidelines [37], p16 IHC was a widely and reliable alternative biomarker. The role of human papillomavirus type was important, but HPV16 was the most common type, accounting for at least 85% of all HPV+ OPSCCs [38], and the number of patients tested for p16 in this study was small (a total of 57), so the number of people who may be infected with non-HPV16 types (such as HPV33) was even smaller, which unlikely change the final statistical results. In addition, the AJCC/UICC 8th Edition New Staging Rules recommended using p16 IHC only as a surrogate for HPV status and divided oropharyngeal carcinomas patients into p16+ patients and p16− ones, so in the actual process of diagnosis and treatment, doctors do not have to identify the HPV type. But further HPV genotyping should be required to determine HPV types when the patient's condition is found to be out of line with the rule of change during treatment.

## Conclusion

Dynamic SII values represent more comprehensive prognostic indicators for patients with oropharyngeal cancer, especially in HPV− patients. In summary, we strongly recommend that doctors consider the dynamic change of SII when making clinical decisions and management, which means that frequent monitoring of SII after radical resection or curative RT/CCRT could provide a useful hint of poor prognosis and help to make individualized therapy. For instance, a patient who experienced unfavorable dynamic changes in SII should be given more frequent image scans or other tests and more advanced treatment to detect and control micro-recurrence lesions as early as possible. In contrast, a patient who experienced favorable dynamic changes can continue with his routine follow-up tests.

## Data Availability

The datasets used and/or analyzed during the current study are available from the corresponding author on reasonable request.

## Abbreviations

SII: Systemic immune inflammation index
HPV: Human papillomavirus
OPC: Oropharyngeal cancer
SCCHN: Squamous cell carcinoma of the head and neck
CBC: Complete blood count
PLT: Platelets
DFS: Disease-free survival
OS: Overall survival
RT: Radiotherapy
CCRT: Chemoradiotherapy
SE: Standard error
HR: Hazard ratio
CI: Confidence interval
SD: Standard deviation
UVA: Univariate Cox analysis
MVA: Multivariate Cox analysis
